# Effect of high-frequency alternating current transcutaneous stimulation over muscle strength: a controlled pilot study

**DOI:** 10.1186/s12984-018-0443-2

**Published:** 2018-11-12

**Authors:** Diego Serrano-Muñoz, Juan Avendaño-Coy, Cristina Simón-Martínez, Julian Taylor, Julio Gómez-Soriano

**Affiliations:** 1 Sensorimotor Function Group, Hospital Nacional de Parapléjicos, 45071 Toledo, Spain; 2 Toledo Physiotherapy Research Group (GIFTO), Nursing and Physiotherapy School, Castilla La Mancha University, 45071 Toledo, Spain; 3 Department of Rehabilitation Sciences, KU Leuven - University of Leuven, 3000 Leuven, Belgium

**Keywords:** Electric stimulation, High-frequency alternating current, Nerve conduction, Motor nerve block, Hand strength

## Abstract

**Background:**

High-frequency alternating currents of greater than 1 kHz applied on peripheral nerves has been used in animal studies to produce a motor nerve block. It has been evidenced that frequencies higher than 5 kHz are necessary to produce a complete peripheral nerve block in primates, whose nerve thickness is more similar to humans. The aim of the study was to determine the effect on muscle strength after the application of a high-frequency stimulation at 5 and 10 kHz compared to sham stimulation in healthy volunteers.

**Findings:**

Transcutaneous stimulation at 5 kHz, 10 kHz and sham stimulation were applied to eleven healthy volunteers over the ulnar and median nerves for 20 min. Maximal handgrip strength was measured before, during, immediately after the intervention, and 10 min after the end of intervention. The 10 kHz stimulation showed a lower handgrip strength during the intervention (28.1 N, SEM 3.9) when compared to 5 kHz (31.1 N, SEM 3.6; *p* < 0.001) and to sham stimulation (33.7 N, SEM 3.9; *p* < 0.001). Furthermore, only stimulation at 10 kHz decreased handgrip strength when compared to baseline.

**Conclusions:**

These findings suggest high-frequency stimulation has an inhibitory effect over muscle strength. Future studies are required in patients that are characterized by motor hyperactive such as spasticity or tremors.

**Clinical trial registration:**

NCT, NCT03169049. Registered on 30 May 2017

## Background

Previous studies in animals have shown that high-frequency alternating current (HFAC) of greater than 1 kHz applied on exposed peripheral nerves can produce a motor nerve block (Bhadra and Kilgore [1, 2]). An in vivo study, [3] showed that frequencies higher than 5 kHz were able to block nerve conduction of motor fibers. One study [4] in non-injured subjects showed an incomplete block when transcutaneous HFAC applied to the radial nerve at 5 kHz increased somatosensory thresholds. It has been evidenced [5] that frequencies higher than 5 kHz are necessary to produce a complete peripheral nerve block in primates, whose nerve diameter is similar to humans, however, there is not any human study that apply HFAC transcutaneously with frequencies higher than 5 kHz. It is believed that the nerve conduction block produced by application of HFAC could be a useful tool for the treatment of patients with pain or with an exaggerated increase of nerve activity, such as hypertonia or spasms.

The purpose of this study was to determine the effects on maximal handgrip strength (MHS) of a non-invasive HFAC at 5 kHz and 10 kHz applied to the ulnar and median nerves in healthy subjects, compared to a sham stimulation.

## Methods

A randomized, crossover, single-blinded, placebo-controlled trial was conducted in 11 healthy volunteers after signing the informed consent approved by the Local Ethics Committee. Participants received three randomized (www.randomizer.org) interventions (10 kHz, 5 kHz, and sham stimulation) with a washout period of 24-h. Moreover, four measurements were registered: before (0 min); during the intervention at 15 min from onset (15 min); immediately after intervention (20 min) and 10 min after the intervention had finished (30 min).

All interventions were applied for 20 min with two surface self-adhesive electrodes (ValuTrode, Axelgaard Manufacturing, USA) 5 cm × 5 cm, which were placed on the anterior face of the dominant forearm, over the ulnar and median nerves. The proximal electrode was fixed in the path of the ulnar nerve over the epitrochlea and the distal electrode was placed on the median nerve over the carpal tunnel. A stimulator (Myomed 932. Enraf Nonius, Netherlands) delivered a sinusoidal current without modulation, at a frequency of 10 kHz. Intensity was determined by a “strong but comfortable tingle” sensation, just below motor threshold. The intensity was gradually increased until a minimally visible contraction was observed and subsequently decreased until it disappeared, and this sensation remained throughout the session. To avoid habituation to the stimulus, participant were asked every 2 minutes to corroborate the perceived sensation, and the intensity was increased if requested [6, 7]. The same procedure was performed with the 5 kHz stimulation. The sham stimulation session was applied by progressively increasing the intensity of a non-connected channel. Participants were also blinded to the hypothesis of the study by being informed that in some cases the perceived sensation might be different because the intensity could be adjusted to below their sensitive threshold, with the possibility that the participant may or may not feel the current [4, 8].

Dynamometry is a reliable and objective method to quantify manual grip strength. The absence or decrease of muscle contraction is considered as an indicator of motor nerve block [8], so dynamometry can be used as an indirect measure to assess the block of motor fibers in humans. Handgrip strength is mediated by ulnar and median nerves and was measured with a handgrip dynamometer (Grip Strength Dynamometer, Tokyo). Three repetitions were registered, and the mean was taken as a value of the average maximal strength.

Statistical analysis was performed with the software “SPSS Statistics 22.0”. Due to the confirmed normal distribution of the data, a parametric test was adopted. Two-way repeated measures ANOVA (time and intervention factors) was performed with a Bonferroni post-hoc test. *p* < 0.05 was considered significant.

## Results

**Fig. 1:**
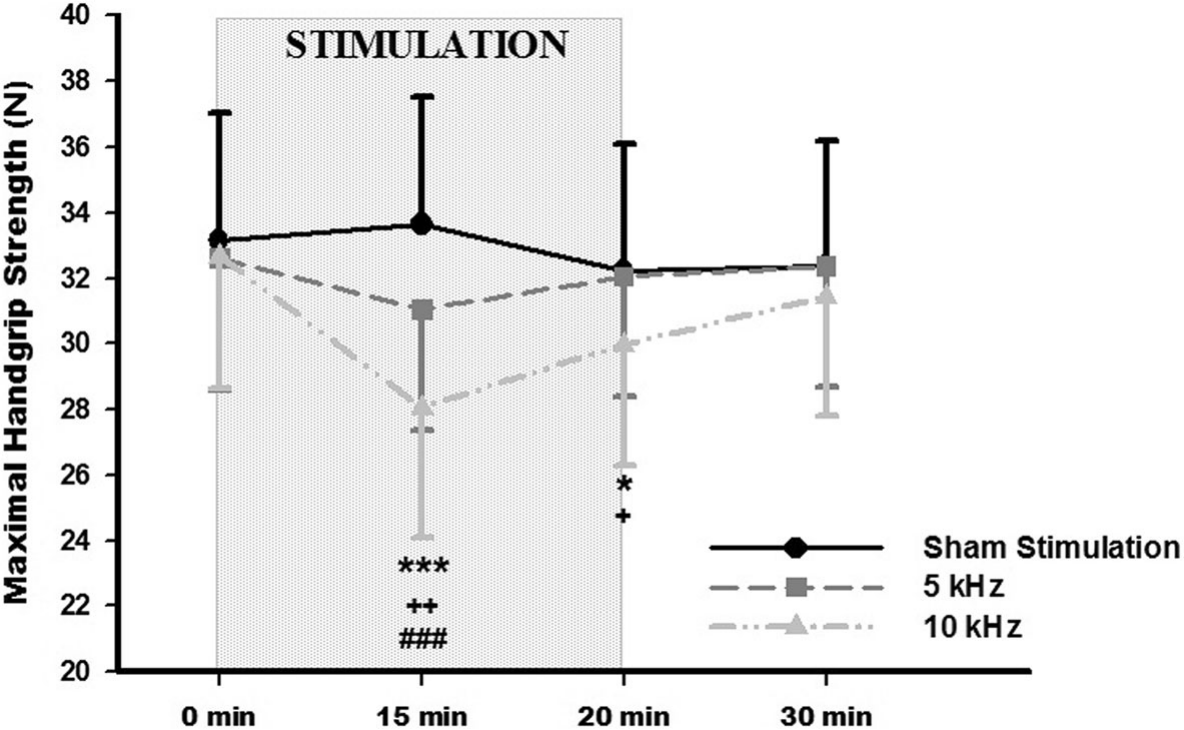
Stimulation effect on maximal handgrip strength. Sham stimulation (circle), 5 kHz (square), and 10 kHz (triangle). Data are represented as mean and standard error. * Indicates significantly different compared to sham stimulation (***p < 0.001; **p < 0.01). ^+^ Indicates a significant difference compared to 5 kHz stimulation (^++^*p* < 0.01). ^#^ Indicates significantly different from baseline (^###^*p* < 0.001)

Eleven subjects (4 males, 36%) with a mean age of 22 years (SEM 1.9) completed the study. The 10 kHz intervention showed a greater initial and final intensity (Initial: 23 mA, SEM 3.3; Final: 39.9 mA, SEM 4.0) than the 5 kHz intervention (Initial: 13.0 mA, SEM 1.4; Final: 21.5 mA, SEM 2.1), *p* < 0.01 and *p* < 0.001, respectively. Table 1 shows data of MHS measurement of each participant. No differences were reported for maximal handgrip strength before the interventions. Significant differences in the “time” factor (F_(3,8)_ = 4.49; *p* = 0.04), the “intervention” factor (F_(2,9)_ = 8.38; *p* = 0.009) and the “time-intervention” interaction (F_(6,5)_ = 6,08; *p =* 0.03) were detected. Specifically, HFAC applied at 10 kHz evidenced a statistically significant decrease in MHS at 15 min during the intervention (28.1 N, SEM 3.9; *p =* 0.001) when compared to baseline (32.6 N, SEM 4.0) and to 10 min post-stimulation (31.4 N, SEM 3.7; *p* = 0.02). Sham stimulation and 5 kHz interventions did not show any statistical change from baseline. Between-group comparisons showed a lower handgrip strength with 10 kHz (28.1 N, SEM 3.9) when compared to 5 kHz (31.1 N, SEM 3.6; *p =* 0.001) and to sham intervention (33.7 N, SEM 3.9; *p =* 0.003) during the stimulation. Immediately after the intervention, a lower MSH was also found in the 10 kHz group (29.9 N, SEM 3.7) when comparing to 5 kHz (32.1 N, SEM 3.6; *p* = 0.02) and sham stimulation (32.2 N, SEM 3.8; *p* = 0.03) (Fig. 1).

**Table 1:** Maximal handgrip strength measurement of each participant throughout the experimental sessions

	10 kHz (Newtons)				5 kHz (Newtons)				Sham Stimulation (Newtons)			
	0 min	15 min	20 min	30 min	0 min	15 min	20 min	30 min	0 min	15 min	20 min	30 min
Participant 1	48.7	43.3	49.5	49.5	54.8	45.1	52.2	50.7	53.9	54.4	51.5	51.1
Participant 2	30.6	25.8	24.7	27.9	27.5	29.3	29.0	29.3	31.8	31.2	31.4	32.3
Participant 3	41.0	35.7	38.1	39.3	39.3	38.9	36.1	40.2	39.4	36.7	39.8	41.9
Participant 4	43.7	35.4	37.0	39.6	45.5	37.1	40.7	41.5	42.0	45.3	42.1	41.4
Participant 5	25.6	18.8	25.2	23.1	24.1	22.9	24.0	24.0	26.6	27.8	25.7	25.3
Participant 6	33.8	32.6	31.2	33.8	34.5	36.4	33.3	30.0	33.4	35.0	35.3	33.5
Participant 7	14.9	15.1	16.2	15.9	16.0	16.5	17.4	17.5	14.7	17.7	14.3	15.4
Participant 8	29.3	25.6	24.8	27.4	28.1	28.9	28.1	30.1	29.6	27.6	26.8	27.9
Participant 9	55.3	52.1	49.5	50.8	50.3	52.6	51.4	52.2	52.2	53.7	50.8	50.4
Participant 10	19.1	14.3	16.4	19.2	17.2	16.9	19.3	18.1	20.6	19.3	17.3	18.1
Participant 11	17.2	10.0	17.3	19.2	21.5	16.9	21.0	22.3	20.3	21.3	19.5	18.9
**Mean**	**32.7**	**28.1**	**30.0**	**31.4**	**32.6**	**31.0**	**32.0**	**32.4**	**33.1**	**33.6**	**32.2**	**32.4**
SD	13.3	13.1	12.2	12.2	13.3	12.1	12.1	12.2	12.8	12.9	12.9	12.6
SEM	4.0	4.0	3.7	3.7	4.0	3.7	3.6	3.7	3.9	3.9	3.9	3.8

Data are expressed in Newtons. *SD* Standard deviation, *SEM* standard error of the mean

## Discussion

This is the first study that applies transcutaneously HFAC at 10 kHz over a human peripheral nerve and a motor nerve conduction block is suggested evidenced by a decrease of MSH. Although there is a high variability among participants, the mean difference of handgrip strength among temporal points have a low variability. This high variability is due to the fact that males have higher levels of handgrip strength than females [9]. The higher intensity needed for the 10 kHz compared to the 5 kHz intervention to achieve the same current perception was expected because higher frequencies have less resistance and less perception of the electrical current [10].

These results are in consonance with experimental animal studies which have shown a peripheral motor conduction block with frequencies between 3 and 10 kHz [11–13]. However, only one study in humans [4] has previously applied transcutaneous HFAC at 5 kHz, with similar effects than conventional TENS on somatosensory thresholds assessment. The higher effect of the 10 kHz current compared to the 5 kHz confirms a frequency-dependent effect suggested by a previous study where the relationship between nerve diameter and block frequency was determined [5]. A key point to note is that the decrease of MHS evidenced during stimulation is quickly reversible when the stimulation ends, as well as the nerve conduction block observed in animals [14]. This could have relevant clinical applications to reduce specific motor activity such as blocking specific nerves during disabling spams or tremors. However, these effects should be studied in subjects with disorders characterized by motor hyperactivity.

### Study limitations

This is a pilot study that assesses handgrip strength as an indirect indicator of motor fiber conduction. However, it is necessary to confirm these results using direct measures such as neurophysiological tests applied in both, healthy and pathological subjects. The major challenge of using neurophysiological tests during the application of HFAC is the artefact evoked by the electrical stimulation.

## Conclusions

High-frequency alternating current stimulation without modulation, at a frequency of 10 kHz applied over ulnar and median nerves of non-injured volunteers produces, during and immediately after the stimulation, a decrease of MHS when compared to HFAC at 5 kHz and sham stimulation. This reduction in MHS could suggest a block of motor nerve activity that could be useful in subjects with neurological disorders characterized by motor hyperactivity.

## Abbreviations

ANOVA: Analysis of variance
HFAC: High-frequency alternating current
MSH: Maximal handgrip strength
N: Newtons
SEM: Standard error mean
